# Comparison of Unilateral and Bilateral Percutaneous Vertebroplasty for Osteoporotic Vertebral Compression Fractures: A Prospective Observational Comparative Study

**DOI:** 10.7759/cureus.105486

**Published:** 2026-03-19

**Authors:** Parthasaradhi Reddy, Ravikumar Biradar, Ashok Nayak

**Affiliations:** 1 Orthopedics, Shri BM Patil Medical College Hospital and Research Centre, BLDE (Deemed to be University), Vijayapura, IND

**Keywords:** bilateral approach, osteoporotic vertebral compression fracture, percutaneous vertebroplasty, unilateral approach, vertebral augmentation

## Abstract

Introduction

Osteoporotic vertebral compression fractures (OVCFs) are a common and debilitating consequence of osteoporosis, frequently resulting in severe back pain, spinal deformity, and functional limitation. Percutaneous vertebroplasty (PVP) is widely used for rapid pain relief and vertebral stabilization. However, the debate persists regarding the optimal surgical approach, unilateral versus bilateral. The study aimed to compare the clinical outcomes, radiological parameters, procedural efficiency, and safety profiles of unilateral and bilateral percutaneous vertebroplasty in patients with osteoporotic vertebral compression fractures.

Materials and methods

This prospective observational comparative study included 40 patients with symptomatic OVCFs, with 20 patients undergoing unilateral PVP and 20 undergoing bilateral PVP based on the operating surgeon’s clinical judgment and institutional practice rather than random allocation. Baseline demographic and clinical characteristics were compared between groups. Outcome measures included operative duration, cement volume, fluoroscopy time, visual analog scale (VAS) scores at multiple follow-up intervals, vertebral height restoration, kyphotic angle correction, and complications.

Results

The unilateral PVP group demonstrated significantly shorter operative time (40.00±2.99 vs 53.65±3.39 minutes), lower cement volume (4.03±0.50 vs 5.03±0.64 mL), and reduced fluoroscopy time (48.10±5.68 vs 70.30±5.68 seconds) compared to the bilateral group (p<0.001 for all). Both groups showed a substantial reduction in VAS scores from a severe baseline (8.00±0.80) to minimal pain at 6 months, with bilateral PVP providing better pain relief at 24 hours (2.40±0.50 vs 3.05±0.83; p=0.005), but no significant differences at later follow-up. Postoperative vertebral height restoration and kyphotic angle correction were comparable between groups (p>0.05). Cement leakage was observed in one (5.0%) patient in the bilateral group and 0 (0.0%) in the unilateral group, with no neurological complications reported.

Conclusion

Unilateral and bilateral PVP provide equivalent long-term clinical and radiological outcomes in OVCFs. The unilateral approach offers improved procedural efficiency without compromising safety or effectiveness.

## Introduction

Osteoporotic vertebral compression fractures (OVCFs) represent one of the most common and clinically significant complications of osteoporosis, contributing substantially to morbidity, reduced quality of life, and increased healthcare utilization worldwide [1]. Osteoporosis affects approximately 200 million individuals globally, with vertebral compression fractures being its most frequent manifestation [1]. The incidence rises markedly with advancing age, particularly among postmenopausal women and elderly men, and nearly 25% of women over 50 years are estimated to sustain at least one vertebral compression fracture during their lifetime [2]. Clinically, OVCFs may range from asymptomatic radiological findings to severe axial back pain, progressive kyphotic deformity, height loss, and functional disability [3]. Persistent pain and immobility not only accelerate further bone loss but also predispose patients to pulmonary compromise, gastrointestinal dysfunction, psychological distress, loss of independence, and increased mortality [3].

The pathophysiology of OVCFs is intrinsically linked to osteoporosis, a metabolic bone disorder characterized by diminished bone mass and microarchitectural deterioration resulting from an imbalance between osteoclastic resorption and osteoblastic formation [3,4]. Vertebral bodies, rich in trabecular bone and exhibiting high metabolic turnover, are particularly vulnerable to structural weakening [5]. Progressive thinning and disruption of the trabecular network reduce the vertebral body's capacity to withstand axial loads, leading to collapse even under minimal trauma [3,6]. Anterior vertebral wedging commonly occurs due to increased compressive forces during flexion [7]. Diagnosis is based on clinical evaluation supplemented by imaging modalities such as plain radiography, computed tomography, and magnetic resonance imaging, the latter being particularly valuable for identifying acute fractures and associated complications [8].

Although conservative management, including analgesics, bracing, immobilization, and anti-osteoporotic therapy, remains the first-line approach, its limitations include inadequate pain control, prolonged immobilization, and risk of medical complications [9]. These challenges have led to the increasing adoption of minimally invasive vertebral augmentation techniques such as percutaneous vertebroplasty [10]. First described by Galibert and Deramond in 1987, vertebroplasty involves percutaneous injection of polymethylmethacrylate cement into the fractured vertebral body to achieve mechanical stabilization and rapid pain relief [11]. Pain reduction is attributed to stabilization of microfractures and thermal and chemical effects on nociceptive fibres [12]. A key technical consideration is the choice between unilateral and bilateral transpedicular approaches. While the bilateral technique may provide more uniform cement distribution, it potentially increases operative time, radiation exposure, and procedural costs, whereas the unilateral approach offers procedural simplicity and reduced exposure, though concerns persist regarding cement distribution and biomechanical stability [13].

Therefore, the present study aimed to compare unilateral and bilateral percutaneous vertebroplasty in patients with osteoporotic vertebral compression fractures, specifically evaluating clinical outcomes (visual analogue scale pain scores), procedural parameters (operative time, cement volume, and fluoroscopy exposure), radiological outcomes (vertebral height restoration and kyphotic angle correction), and procedure-related complications in a prospective observational comparative study design.

This research work was originally conducted as part of a postgraduate dissertation submitted to the Department of Orthopedics of BLDE (Deemed to be University), Vijayapura, Karnataka, India.

## Materials and methods

Study design and setting

This prospective observational comparative study was conducted in the Department of Orthopedics at BLDE (Deemed to be University), Shri BM Patil Medical College Hospital and Research Centre, Vijayapura, Karnataka, from June 2024 to December 2025.

Ethics statement

Ethical approval was obtained from the Institutional Ethics Committee of BLDE (Deemed to be University) (IEC No: BLDE(DU)/IEC-SBMPMC/140/2023-24). The study was conducted in accordance with the Declaration of Helsinki, and written informed consent was obtained from all participants prior to enrolment.

Study population and sample size

Sample size estimation was performed using G*Power version 3.1.9.4 based on previously published operative time differences reported by Liu et al., which demonstrated significantly shorter operative duration in unilateral compared to bilateral vertebroplasty (40.5 ± 9.0 vs. 57.2 ± 11.4 minutes) [14]. Considering this effect size, with 80% power and a 1% alpha error using a two-tailed test, the minimum required sample size was 36. To enhance statistical robustness and compensate for potential attrition, 40 patients were enrolled in the study, of whom 20 underwent unilateral vertebroplasty, and 20 underwent bilateral vertebroplasty.

Adult patients aged above 40 years with radiologically confirmed osteoporotic vertebral compression fractures and persistent back pain despite adequate conservative management, with a Visual Analogue Scale (VAS) score ≥5, were included. Patients with pathological fractures secondary to malignancy, spinal infections, cervical or sacral fractures, neurological deficits requiring decompression, age below 40 years, or VAS score <5 were excluded.

Study measures and preoperative evaluation

Patients underwent either unilateral or bilateral percutaneous vertebroplasty based on the operating surgeon's clinical judgment, fracture characteristics, and standard institutional practice; therefore, patient allocation was not randomized. Baseline demographic details, clinical characteristics, and pain severity using the visual analog scale (VAS) were recorded prior to the procedure to assess comparability between the two groups.

Preoperative evaluation included detailed clinical assessment, neurological examination, routine hematological and biochemical investigations, and radiological assessment with anteroposterior and lateral thoracolumbar spine radiographs. Magnetic resonance imaging was performed to confirm acute osteoporotic fractures and exclude alternative pathologies, while computed tomography was obtained when necessary for better delineation of fracture morphology and pedicle anatomy.

Surgical procedure

All procedures were performed under general anesthesia with patients positioned prone on a radiolucent operating table under fluoroscopic guidance. In the unilateral approach, a single transpedicular needle was advanced across the midline into the anterior third of the vertebral body. In the bilateral approach, needles were inserted through both pedicles and positioned at the anterior-middle third junction of the vertebral body.

Polymethylmethacrylate cement was prepared according to manufacturer recommendations and injected under continuous fluoroscopic monitoring in anteroposterior and lateral views. Cement injection was terminated upon achieving satisfactory vertebral filling or detection of leakage. Operative time, cement volume injected, fluoroscopy duration, and intraoperative complications were recorded for each procedure.

Follow-up and outcome measures

**Figure 1:**
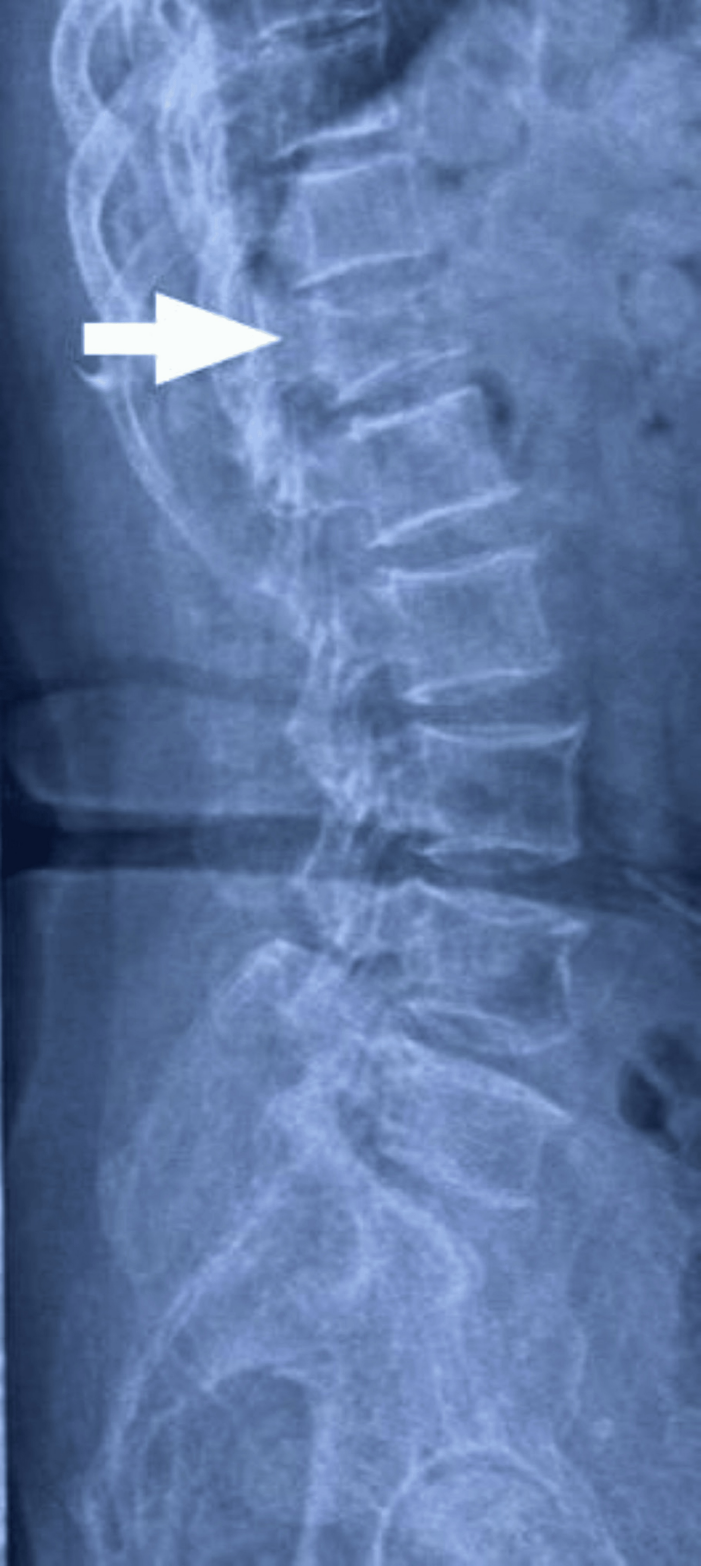
X-ray of skeletally mature patient showing lumbosacral spine lateral view with D12 wedge compression fracture

**Figure 2:**
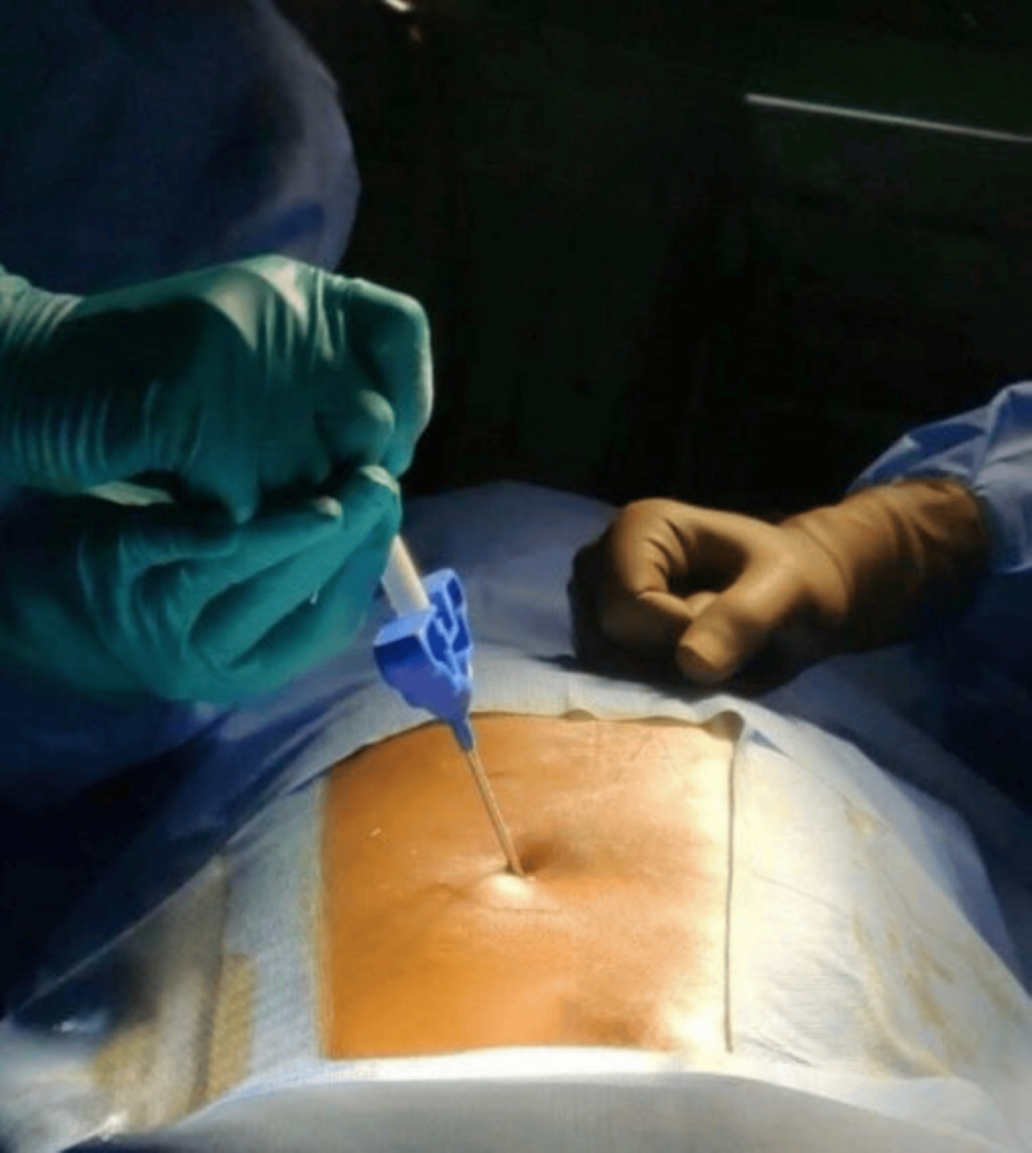
Intraoperative image of injecting bone cement through Jamshidi needle (unipedicular approach)

**Figure 3:**
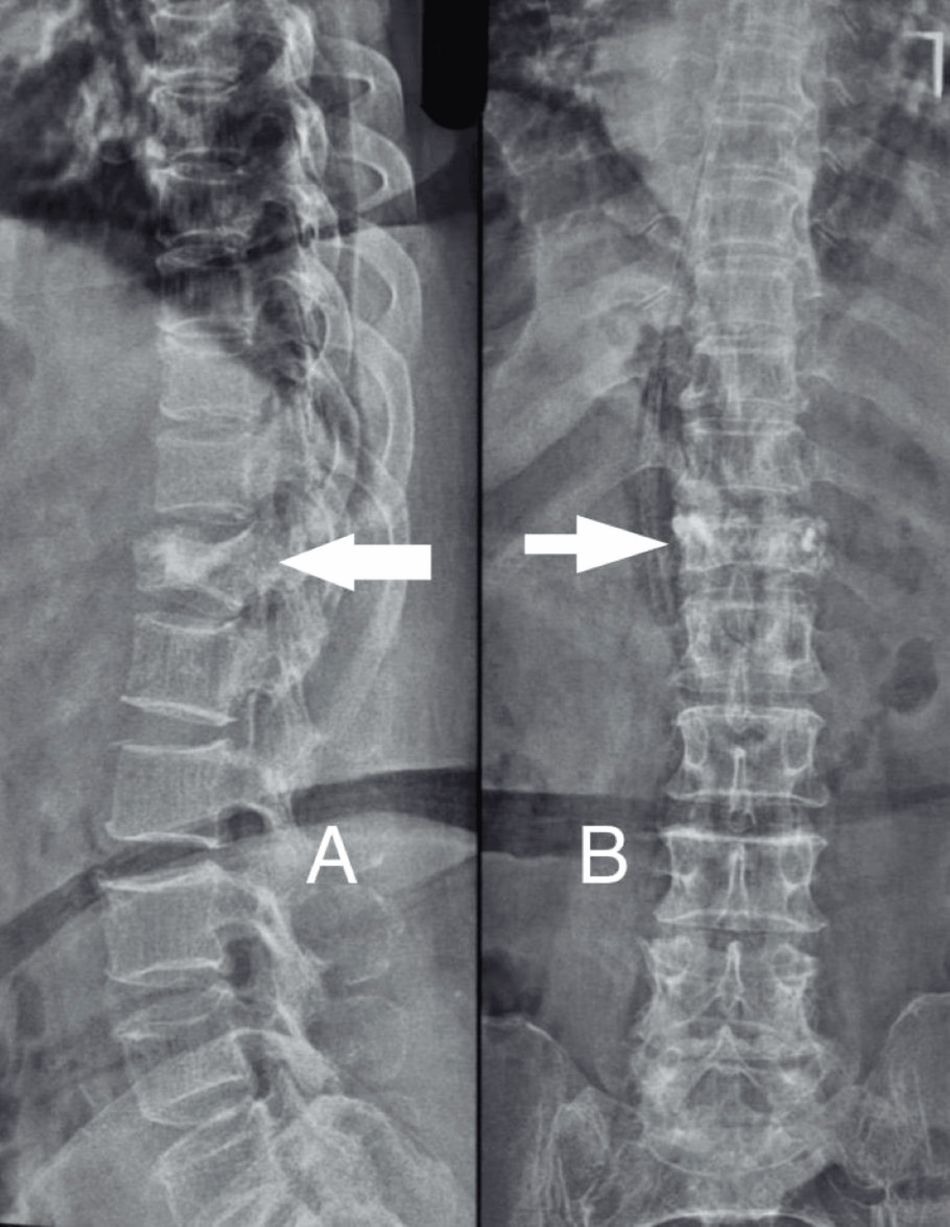
A) postoperative X-ray of same patient showing Lumbosacral spine lateral view with bone cement in D12 vertebrae; B) postoperative X-ray of same patient showing lumbosacral spine anteroposterior view with bone cement in D12 vertebrae

**Figure 4:**
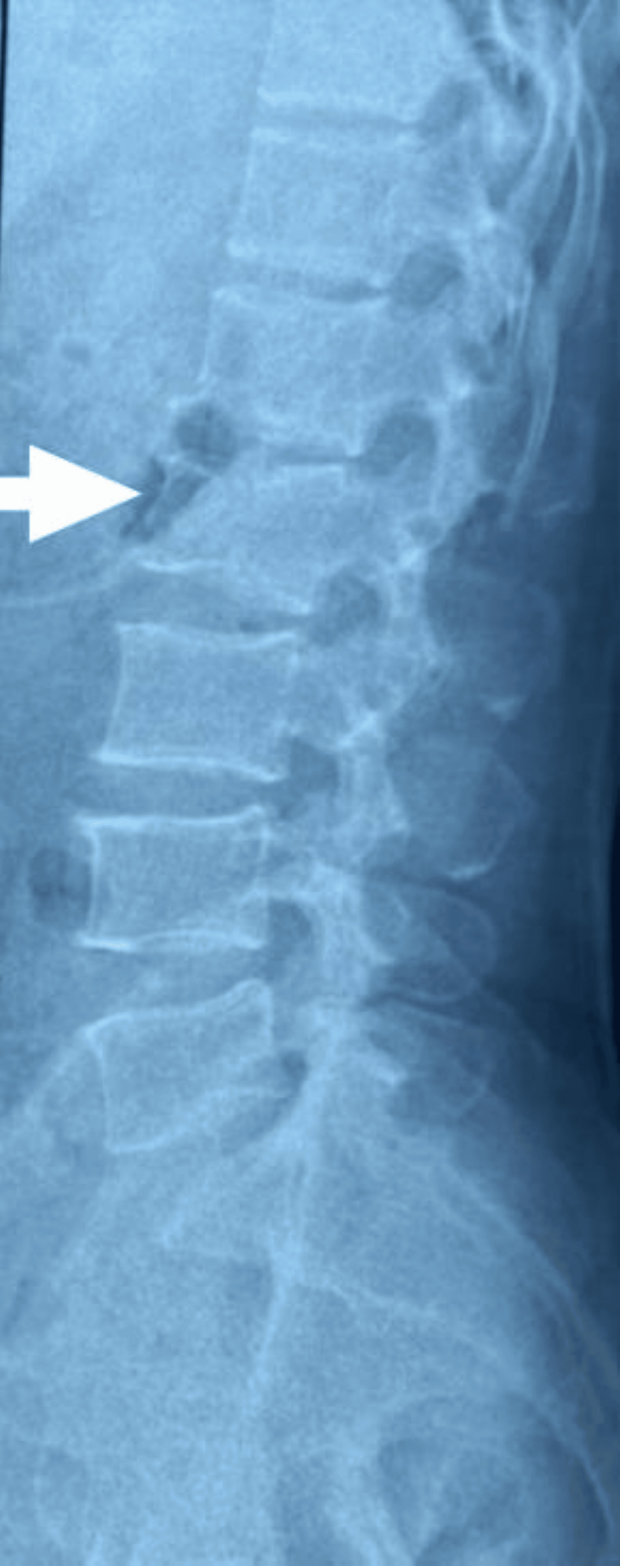
X-ray of skeletally mature patient showing lumbosacral spine lateral view with L4 wedge compression fracture

**Figure 5:**
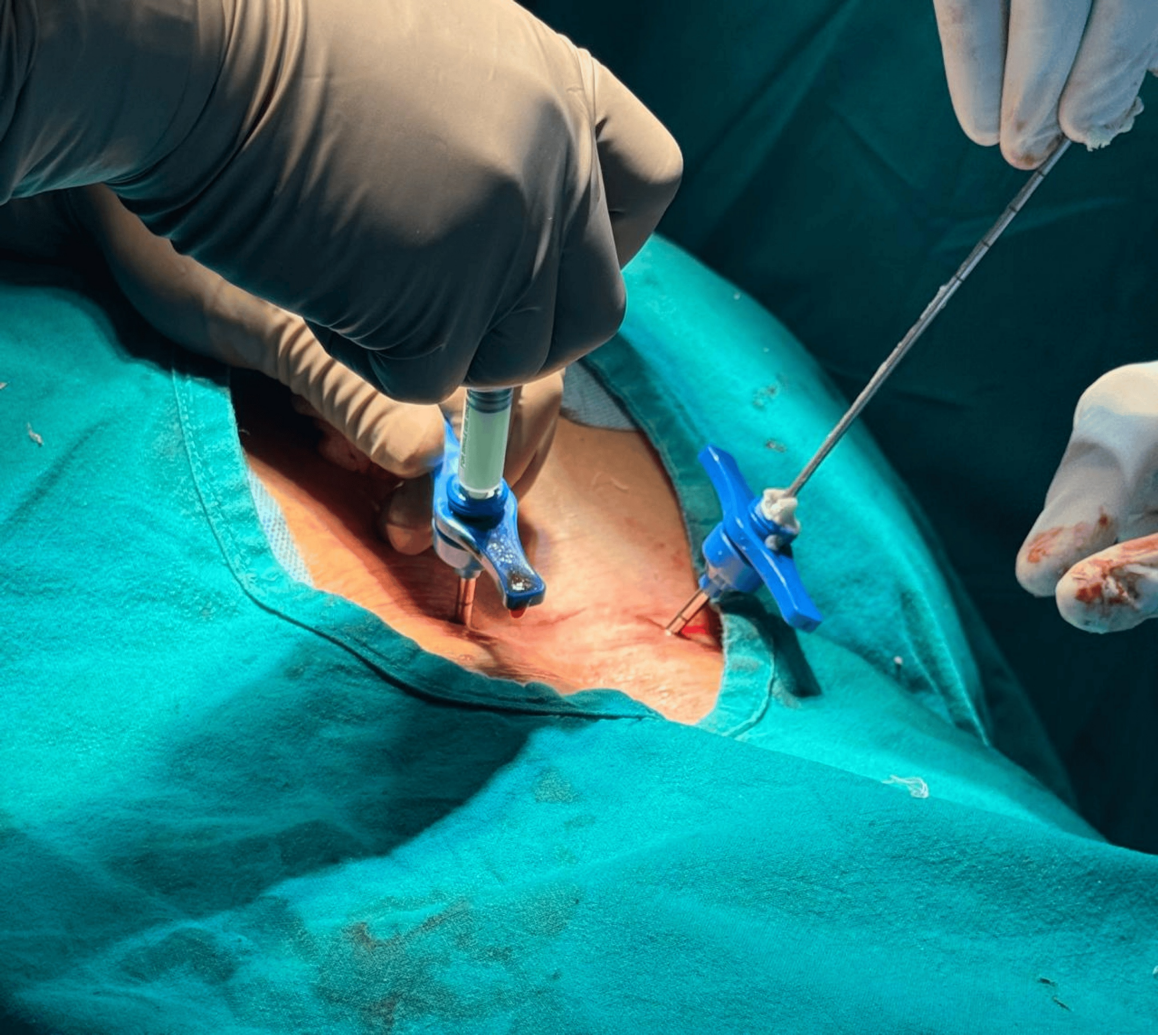
Intraoperative image of injecting bone cement through Jamshidi needle (bipedicular approach)

**Figure 6:**
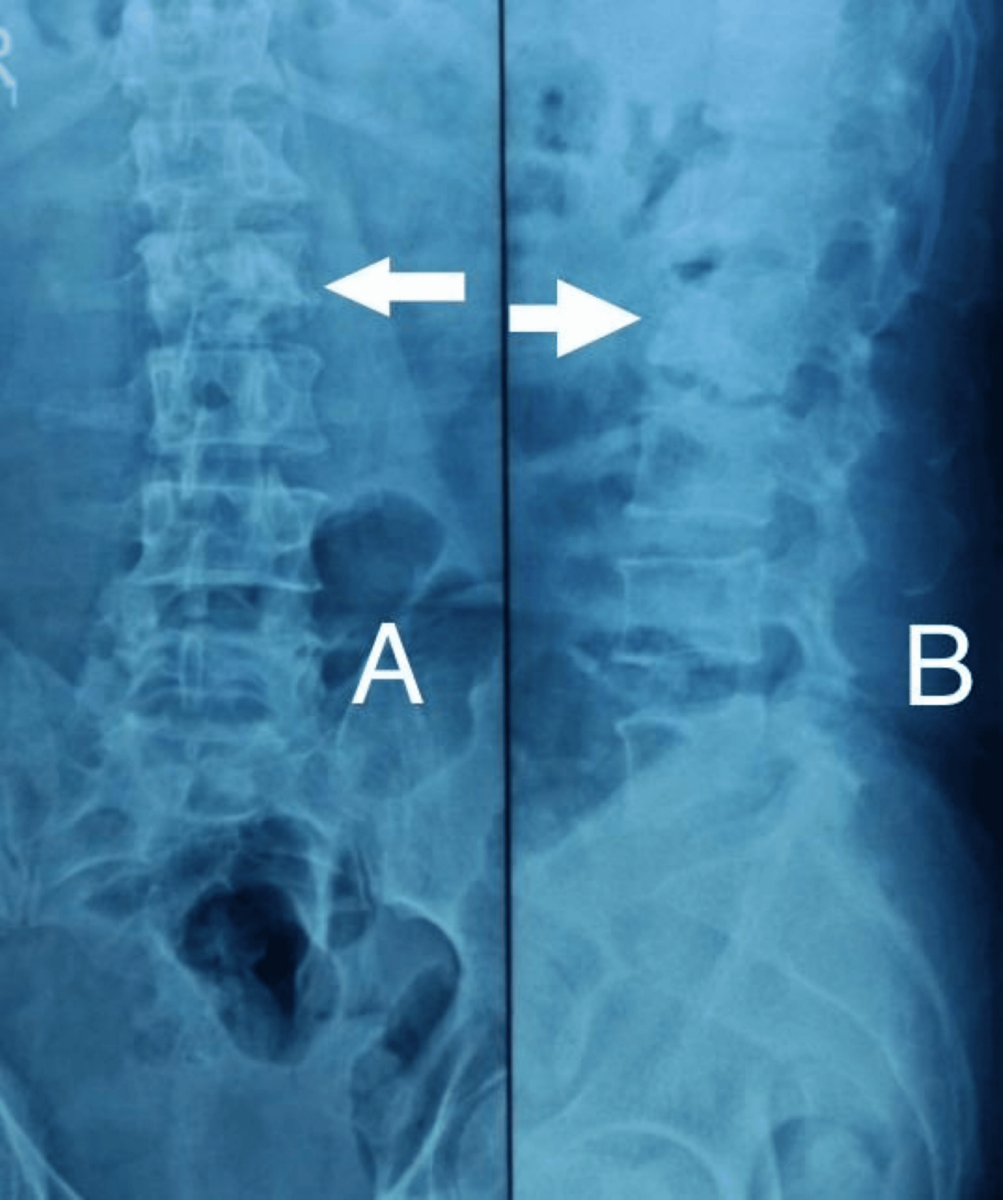
A) postoperative X-ray of same patient showing lumbosacral spine anteroposterior view with bone cement in L4 vertebrae; B) postoperative X-ray of same patient showing lumbosacral spine lateral view with bone cement in L4 vertebrae

Postoperative neurological evaluation and radiographic assessment were performed to confirm cement distribution and detect extravasation. Early mobilization was encouraged once patients were clinically stable. Follow-up evaluations were conducted at three weeks, six weeks, three months, and six months post-procedure. Outcome measures included VAS pain score, radiographic vertebral height restoration, kyphotic angle correction, cement distribution pattern, development of adjacent-level fractures, and complications. Radiological measurements, including vertebral height restoration and kyphotic angle, were assessed using standardized measurements on anteroposterior and lateral thoracolumbar spine radiographs obtained during follow-up (Figure 1-6).

Statistical analysis

Statistical analysis was performed using SPSS v26 (IBM Inc., Armonk, New York). Continuous variables were analyzed using an independent samples t-test or Mann-Whitney U test as appropriate, and categorical variables were compared using Chi-squared or Fisher's exact test. A p-value of <0.05 was considered statistically significant.

## Results

The two groups were comparable with respect to baseline demographic and clinical parameters. Most patients were aged 61-80 years (bilateral: 16 (80.0%); unilateral: 15 (75.0%); p=1.000), with similar mean age (67.30±7.37 vs 66.90±6.82 years; p=0.860). Female predominance was observed in both groups (bilateral: 16 (80.0%); unilateral: 13 (65.0%); p=0.287). Mean BMI did not differ significantly (23.60±1.27 vs 23.45±1.16 kg/m²; p=0.708). Hypertension was the most frequent comorbidity (seven (35.0%) in both groups; p=0.813). Preoperative pain severity was identical (VAS 8.00±0.80 in both groups; p=1.000), as were vertebral height loss (36.60±4.16%; p=1.000) and kyphotic angle (19.55±3.56°; p=1.000), confirming homogeneity between groups prior to intervention (Table 1).

**Table 1:** Baseline demographic and preoperative clinical characteristics Data are presented as frequency with percentage for categorical variables and mean ± SD for continuous variables. Comparisons between bilateral PVP and unilateral PVP groups were performed using the Chi-squared (χ²) test for categorical variables and the independent sample t-test for continuous variables. PVP - percutaneous vertebroplasty; BMI - body mass index; VAS - visual analogue scale; HTN - hypertension; DM - diabetes mellitus; SD - standard deviation

Parameter		Bilateral PVP (n=20)	Unilateral PVP (n=20)	Test statistic	p-value
Age	40-60 years	4 (20.0%)	5 (25.0%)	χ² = 0.13	1.000
	61-80 years	16 (80.0%)	15 (75.0%)		
	Mean ± SD	67.30 ± 7.37	66.90 ± 6.82	t = 0.18	0.860
Gender	Male	4 (20.0%)	7 (35.0%)	χ² = 1.14	0.287
	Female	16 (80.0%)	13 (65.0%)		
BMI (kg/m²)	Mean ± SD	23.60 ± 1.27	23.45 ± 1.16	t = 0.38	0.708
Comorbidities	Hypertension	7 (35.0%)	7 (35.0%)	χ² = 0.89	0.813
	Diabetes Mellitus	3 (15.0%)	2 (10.0%)		
	HTN + DM	1 (5.0%)	3 (15.0%)		
	None	9 (45.0%)	8 (40.0%)		
Preoperative VAS score	Mean ± SD	8.00 ± 0.80	8.00 ± 0.80	t = 0.01	1.000
Preoperative vertebral height loss (%)	Mean ± SD	36.60 ± 4.16	36.60 ± 4.16	t = 0.01	1.000
Preoperative kyphotic angle (°)	Mean ± SD	19.55 ± 3.56	19.55 ± 3.56	t = 0.01	1.000

Significant differences were observed in intraoperative parameters between the two approaches. The unilateral group demonstrated a significantly shorter operative time (40.00±2.99 minutes) compared to the bilateral group (53.65±3.39 minutes; p<0.001). Cement volume was significantly lower in the unilateral group (4.03±0.50 mL) than in the bilateral group (5.03±0.64 mL; p<0.001). Additionally, fluoroscopy time was markedly reduced with the unilateral approach (48.10±5.68 seconds vs 70.30±5.68 seconds; p<0.001), indicating improved procedural efficiency and reduced radiation exposure (Table 2).

**Table 2:** Comparison of intraoperative parameters Data are presented as mean ± SD. Intergroup comparisons between bilateral PVP and unilateral PVP were performed using the independent sample t-test. PVP - percutaneous vertebroplasty; SD - standard deviation; mL - milliliter

Parameter (mean ± SD)	Bilateral PVP (n=20)	Unilateral PVP (n=20)	Test statistic	p-value
Surgery duration (minutes)	53.65 ± 3.39	40.00 ± 2.99	t = 13.62	<0.001
Bone cement volume (mL)	5.03 ± 0.64	4.03 ± 0.50	t = 5.57	<0.001
Fluoroscopy time (seconds)	70.30 ± 5.68	48.10 ± 5.68	t = 12.37	<0.001

Both groups experienced substantial and sustained postoperative pain reduction. Although preoperative VAS scores were identical (8.00±0.80; p=1.000), the bilateral group demonstrated significantly lower pain scores at 24 hours (2.40±0.50 vs 3.05±0.83; p=0.005). However, this early difference was not maintained. At six weeks (2.00±0.73 vs 2.15±0.67; p=0.501), three months (1.80±0.62 vs 1.85±0.67; p=0.807), and six months (0.10±0.45 vs 0.25±0.63; p=0.391), VAS scores were comparable, demonstrating equivalent long-term analgesic efficacy between the two techniques (Table 3).

**Table 3:** Visual analog scale (VAS) scores at different time points Data are presented as mean ± SD. Comparisons between bilateral PVP and unilateral PVP groups at each time point were performed using the independent sample t-test. VAS - visual analog scale; PVP - percutaneous vertebroplasty; SD - standard deviation

Time point	Bilateral PVP (n=20)	Unilateral PVP (n=20)	Test statistic	p-value
Preoperative	8.00 ± 0.80	8.00 ± 0.80	t = 0.00	1.000
24 hours	2.40 ± 0.50	3.05 ± 0.83	t = 3.02	0.005
6 weeks	2.00 ± 0.73	2.15 ± 0.67	t = 0.68	0.501
3 months	1.80 ± 0.62	1.85 ± 0.67	t = 0.25	0.807
6 months	0.10 ± 0.45	0.25 ± 0.63	t = 0.87	0.391

Radiological outcomes were comparable between the two groups. Postoperative vertebral height restoration did not differ significantly (85.70±3.36% in bilateral vs 84.70±3.36% in unilateral; p=0.352). Similarly, postoperative kyphotic angle correction was comparable (11.75±2.34° vs 12.75±2.34°; p=0.184). Cement leakage was observed in one (5.0%) patient in the bilateral group and none in the unilateral group (p=0.310). No neurological complications occurred in either group. Overall, both techniques demonstrated similar radiological effectiveness and safety profiles (Table 4).

**Table 4:** Radiological and safety outcomes Continuous variables are presented as mean ± SD, while categorical variables are presented as frequency with percentage. Intergroup comparisons were performed using the independent sample t-test for continuous variables and the Chi-squared (χ²) test for categorical variables. PVP - percutaneous vertebroplasty; SD - standard deviation

Parameter	Bilateral PVP (n=20)	Unilateral PVP (n=20)	Test statistic	p-value
Postoperative vertebral height restoration (%), mean ± SD	85.70 ± 3.36	84.70 ± 3.36	t = 0.94	0.352
Postoperative kyphotic angle (°), mean ± SD	11.75 ± 2.34	12.75 ± 2.34	t = 1.35	0.184
Cement leakage, n (%)	1 (5.0%)	0 (0.0%)	χ² = 1.025	0.310
Neurological complications, n (%)	0 (0.0%)	0 (0.0%)	-	-

## Discussion

The present study demonstrated that unilateral percutaneous vertebroplasty (PVP) offers significant procedural advantages over the bilateral approach while maintaining comparable clinical and radiological outcomes in this prospective observational comparative study. Operative duration was significantly shorter with the unilateral technique, consistent with findings reported by Liu et al., who observed reduced operative times with unilateral access [14]. Similar results were documented by Zhang et al. and Che et al., supporting the procedural efficiency of unilateral vertebroplasty [15,16]. In elderly patients with osteoporotic vertebral compression fractures, reduced operative time is clinically relevant as it minimizes anesthesia exposure and perioperative risk.

The unilateral group also required significantly lower cement volume and fluoroscopy time. Reduced cement usage aligns with previous observations by Liu et al. and Chen et al., who reported higher cement volumes with bilateral approaches [14,16]. Yan et al. similarly demonstrated increased fluoroscopic exposure during bilateral procedures due to the need for dual pedicle visualization and symmetrical cement confirmation [17]. These findings collectively reinforce the efficiency and safety advantages of the unilateral technique.

Regarding pain outcomes, both approaches resulted in marked and sustained reduction in VAS scores from severe baseline pain to minimal levels at follow-up. Although bilateral PVP demonstrated superior pain relief at 24 hours, this difference was not sustained beyond the immediate postoperative period. At subsequent follow-up intervals, pain scores were comparable between groups, consistent with findings by Sun et al., Rebolledo et al., who reported no significant long-term differences in pain reduction between unilateral and bilateral vertebroplasty [18,19]. These results suggest that once adequate vertebral stabilization is achieved, long-term analgesic outcomes are independent of the pedicular approach.

Radiological outcomes in the present study were comparable between the two techniques, with similar vertebral height restoration and kyphotic angle correction. Biomechanical evidence from Steinmann et al. supports that unipedicular cement augmentation can provide stability equivalent to bipedicular methods when adequate midline cement distribution is achieved [20]. Cement leakage was observed in one (5.0%) patient in the bilateral group and none (0.0%) in the unilateral group, a difference that was not statistically significant. Larger comparative studies by Liu et al., Yan et al., and Chen et al. have reported higher leakage rates with bilateral approaches, potentially related to greater cement volume and increased intravertebral pressure [14,21,22]. Importantly, no neurological complications were observed in either group in the present study, further supporting the safety of both techniques when performed under appropriate fluoroscopic guidance.

While the findings of the present study suggest that unilateral vertebroplasty offers procedural advantages without compromising clinical or radiological outcomes, the results should be interpreted cautiously given the non-randomized observational design and the relatively small sample size.

The present study has several strengths as well as limitations that should be acknowledged. A major strength of this study is its prospective comparative design, which allowed systematic data collection and standardized follow-up assessment of both clinical and radiological outcomes. Additionally, the study evaluated multiple clinically relevant parameters, including pain relief, operative time, fluoroscopy exposure, cement volume, vertebral height restoration, and kyphotic angle correction, thereby providing a comprehensive assessment of procedural effectiveness and safety. However, certain limitations must also be considered. The relatively small sample size (40 patients) may limit statistical power, particularly for detecting differences in infrequent complications such as cement leakage or neurological events. The follow-up duration of six months may be insufficient to evaluate long-term outcomes, including adjacent-level fractures and sustained deformity correction. The single-center design may restrict generalizability to other practice settings, and functional outcome measures such as disability indices or quality-of-life scores were not assessed. Additionally, because patient allocation to unilateral or bilateral procedures was based on surgeon judgment rather than randomization, the possibility of selection bias cannot be completely excluded despite comparable baseline characteristics between groups.

## Conclusions

Both unilateral and bilateral percutaneous vertebroplasty are safe and effective minimally invasive options for the management of osteoporotic vertebral compression fractures, providing sustained pain relief and satisfactory radiological restoration. Although minor differences may exist in early postoperative recovery, long-term clinical and structural outcomes are comparable between the two approaches. The unilateral technique offers practical advantages related to procedural efficiency and reduced intraoperative exposure without compromising therapeutic effectiveness. However, these findings should be interpreted in the context of the study’s non-randomized observational design, relatively small sample size, and limited follow-up duration. Therefore, unilateral vertebroplasty may be considered an appropriate and efficient approach in routine clinical practice, while the choice of technique should ultimately be individualized based on patient characteristics, fracture morphology, and surgeon expertise. Further large-scale randomized studies with longer follow-up are warranted to confirm these findings and strengthen the evidence base.
